# *In Vitro* Effects of Strontium on Proliferation and Osteoinduction of Human Preadipocytes

**DOI:** 10.1155/2015/871863

**Published:** 2015-07-09

**Authors:** V. Nardone, R. Zonefrati, C. Mavilia, C. Romagnoli, S. Ciuffi, S. Fabbri, G. Palmini, G. Galli, A. Tanini, M. L. Brandi

**Affiliations:** Department of Surgery and Translational Medicine, University of Florence, Largo Brambilla 3, 50139 Florence, Italy

## Abstract

Development of tools to be used for *in vivo* bone tissue regeneration focuses on cellular models and differentiation processes. In searching for all the optimal sources, adipose tissue-derived mesenchymal stem cells (hADSCs or preadipocytes) are able to differentiate into osteoblasts with analogous characteristics to bone marrow mesenchymal stem cells, producing alkaline phosphatase (ALP), collagen, osteocalcin, and calcified nodules, mainly composed of hydroxyapatite (HA). The possibility to influence bone differentiation of stem cells encompasses local and systemic methods, including the use of drugs administered systemically. Among the latter, strontium ranelate (SR) represents an interesting compound, acting as an uncoupling factor that stimulates bone formation and inhibits bone resorption. The aim of our study was to evaluate the *in vitro* effects of a wide range of strontium (Sr^2+^) concentrations on proliferation, ALP activity, and mineralization of a novel finite clonal hADSCs cell line, named PA20-h5. Sr^2+^ promoted PA20-h5 cell proliferation while inducing the increase of ALP activity and gene expression as well as HA production during *in vitro* osteoinduction. These findings indicate a role for Sr^2+^ in supporting bone regeneration during the process of skeletal repair in general, and, more specifically, when cell therapies are applied.

## 1. Introduction

The development of alternative treatments for bone tissue regeneration, both in bone fractures and skeletal defects due to trauma, congenital malformations, or tumor excisions, is a rapidly growing area of investigation. Despite its several limitations, bone tissue transplantation at this time represents the treatment of choice for large bone defects [1]. However, in recent years, new strategies for improving bone regeneration have been introduced, and several clinical studies have shown that human mesenchymal stem cells (hMSCs) have the ability to accelerate the healing of bone defects due to their potential to differentiate into osteoblasts [2–6]. These cells, either alone or in combination with biomaterials enriched with osteoinductive factors (i.e., bone morphogenetic proteins (BMPs)), have been demonstrated to promote the formation of bone tissue [7]. Their expansion and multipotential proprieties indicate adult hMSCs as good candidates for regenerative cell therapy of injured bone tissue [8]. Classically, hMSCs are isolated from adult bone marrow aspirates, expanded* ex vivo* [9], and implanted* in vivo* on inorganic biocompatible (ceramic, hyaluronic acid, and synthetic polymers) osteoconductive scaffolds [10, 11].

In addition to bone marrow, adult hMSCs have been identified in several tissues such as adipose tissue, dental pulp, skeletal muscle, umbilical cord, and human amniotic fluid [12]. Human stem cells from connective tissues have the capability to differentiate, under appropriate* in vitro* conditions, into various mesenchymal lineages such as adipocytes, chondrocytes, myocytes, hepatocytes, endothelial cells, hematopoietic cells, neuronal cells, and osteoblasts [13–22]. In particular, human adipose tissue-derived mesenchymal stem cells (hADSCs) have been demonstrated to have the ability to differentiate into functional osteoblasts, like human bone marrow-derived mesenchymal stem cells (hBMMSCs), expressing several osteoblastic phenotypes [23, 24]. When compared to hBMMSCs, hADSCs have been shown to be immunoprivileged [25, 26], with a higher genetic stability in long-term culture [27–29]. These characteristics, together with easier access to large bioptic samples [30–32] and lower invasiveness of tissue sampling when compared to hBMMSCs, make hADSCs an ideal source for bone regeneration [9].

Human ADSCs are capable of secreting a large number of cytokines and growth factors that support angiogenesis, tissue remodelling, and antiapoptotic effects such as VEGF, HGF, Il-6, Il-7, TNF*α*, M-CSF, and TGF-*β*1 [33], all important in the bone regeneration process [30]. The safety and efficacy of hADSCs for tissue reconstruction, as well as for applications in graft-versus-host disease, are currently under assessment in clinical trials [34–38]. Furthermore, these cells are under evaluation for potential use in immunosuppression in various systemic disorders [25, 26, 39, 40] and in soft tissue replacement [41, 42].

The application of cell therapies in bone tissue engineering should also consider the possibility to use systemic drugs capable of modulating bone cell function and proliferation. Indeed, several effective drugs are currently available for the treatment of bone metabolism disorders such as aminobisphosphonates (BPs) [43–45], selective estrogen receptor modulators (SERMs) [46, 47], an antireceptor activator of NFkB ligand (RANKL) monoclonal antibody [48, 49], parathyroid hormone peptides [50, 51], and SR [52–54]. All these compounds are either purely antiresorptive or purely anabolic, but SR, an agent with dual effects on bone remodelling, is able to stimulate bone formation and inhibit bone resorption [55, 56].

SR is composed of two cations of Sr^2+^, which represent the active component, and one anion of ranelate, which acts as carrier [57]. In bone, the majority of the strontium ions are absorbed on the surface of hydroxyapatite crystals and excreted through the kidneys and the faeces, with a preferential distribution in cancellous newly formed bone [58, 59]. In animal studies, an increment in osteoid surface and bone volume and trabecular thickness have been seen after SR treatment [60].

Several findings support an osteogenic role for Sr^2+^, with stimulatory action on osteoprogenitor cell proliferation and differentiation into mature osteoblasts, by induction of osteoblastic proteins expression [22, 61, 62]. Studies on rodent and human primary osteoblast cultures have shown that Sr^2+^, like calcium, acts as an agonist on the calcium-sensing receptor (CaSR), promoting cell replication, differentiation, and survival [63–66]. Moreover, through osteoblastic cell stimulation, Sr^2+^ is able to influence the osteoclastogenesis and the function of mature osteoclasts [63, 67–69].* In vitro* studies in both primary human osteoblastic cells and murine rat calvaria cells have shown that Sr^2+^ induced an increase in mRNA and protein levels of osteoprotegerin (OPG) with suppression of RANKL expression, thus favouring downregulation of osteoblast-induced osteoclastogenesis [64, 70–72].

Altogether, these findings support a role of Sr^2+^ on osteoblastogenesis and bone repair. Indeed, a study on a calvarial defect model in rats has shown that Sr^2+^ enhanced the osteogenic differentiation of the MSCs, upregulating extracellular matrix (ECM) gene expression and stimulating the Wnt/*β*-catenin pathway [73]. Moreover, Sr^2+^ promotes osteogenic differentiation of rat BMMSCs by increasing the expression of bone morphogenetic protein-7 (BMP-7), ALP, Cbfa1/RUNX2, bone sialoprotein, and osteocalcin [74, 75]. Furthermore, an* in vitro* study on mice BMMSCs has demonstrated that Sr^2+^ induces osteoblastic differentiation through induction of prostaglandin E_2_ synthesis [76]. Finally, Sr^2+^ enhances the calcium deposition process and promotes bone repair, through enhancing the osteogenic differentiation of hMSCs [77, 78].

Given the absence of data on the role of Sr^2+^ on hADSCs proliferation and osteoinduction, the aim of this study was to evaluate the* in vitro* action of Sr^2+^ on the molecular mechanisms regulating these processes in a clonal cell line of hADSCs.

## 2. Materials and Methods

### 2.1. Cell Cultures

A hADSCs line, named PA20, was isolated from small fragments of subcutaneous adipose tissue biopsy obtained during orthopedic surgery from a female patient aged 45 years, after signing informed consent in accordance with a protocol approved by the Institutional Review Board for human studies. Briefly, the adipose tissue sample was minced into small pieces (0.2–0.5 mm) and digested for 3 h at 37°C in Ham's F12 Coon's modification medium supplemented with 20% fetal bovine serum (FBS) and 3 mg/mL collagenase type I (C-0130, Sigma-Aldrich). The tissue was then mechanically dispersed by pipetting and passed through a sterile 230 *μ*m stainless steel tissue sieve. The undigested tissue trapped in the sieve was discarded, while the infranatant containing the hADSCs fraction was collected and the cells were sedimented by centrifugation at 300 g for 5 min. The cells were resuspended and cultured in 100 mm tissue culture plates at 37°C in humidified atmosphere with 5% CO_2_ in growth medium (GM); thus Ham's F12 Coon's modification medium supplemented with 10% FBS, 100 IU/mL penicillin, 100 *μ*g/mL streptomycin, and 1 ng/mL basic fibroblast growth factor (bFGF) was composed. The medium was refreshed twice a week and the cells were used for further subculturing or cryopreservation upon reaching 5 × 10^3^ cells/cm^2^.

The human continuous osteoblastic-like cell line SaOS-2, derived from human osteosarcoma, obtained from the American Type Cultures Collection (ATCC, Rockville, MD, USA) was used as positive control. Cells were cultured in GM and differentiated in OM as PA20-h5 cell line.

### 2.2. Cell Cloning

PA20 cells at the 3rd passage were used for cell cloning. Cells in an active phase of growth were cloned by the dilution plating technique. Cells were detached with trypsin 1 : 250 0.4 mg/mL in Dulbecco's phosphate buffered saline (DPBS) without Ca^2+^, without Mg^2+^, with EDTA 0.2 mg/mL, and with glucose 1 mg/mL, resuspended in Coon's medium + 20% FCS. The cell suspension was diluted to a concentration of 10 cells/mL in the following cloning medium: Coon's + 20% FCS supplemented with 25% conditioned medium prepared from human foetal fibroblast culture. The cell suspension was maintained in agitation and 0.1 mL was rapidly distributed per well of a 96-well, half area tissue culture plate. Each well was carefully observed and the wells containing only one cell were scored. The cloning culture was incubated at 37°C in humidified air with 5% CO_2_. When colonies reached the consistency of 500–600 cells, they were detached, collected, and first transferred in 24 multiwell plates and subsequently expanded in 60 mm and 100 mm dishes.

### 2.3. Soft Agar Assay for Neoplastic Transformation

Neoplastic transformed cells form colonies that grow progressively in soft agar. A 35 mm dish was coated with 1% agar prepared in culture medium maintained liquid at 45°C. The dish was immediately cooled. Cells in growth phase were detached, suspended in medium, diluted to double the required final concentration, and maintained at 37°C. 0.68% agar was prepared in medium and maintained at 45°C. Cell suspension was mixed with an equal volume of 0.68% agar, distributed into the agar coated dish to obtain a final concentration of 2.5 × 10^3^ cells/dish, and immediately cooled. The cells were cultured at 37°C in humidified air with 5% CO_2_ for 3-4 weeks until the formation of colonies and their growth. Colonies formed per dish were observed and counted in phase contrast microscopy.

### 2.4. Cell Line Characterization

The characterization of the PA20 cell line and the finite clonal cell line, named PA20-h5, was performed by studying the doubling time, the soft agar assay, and finally both the adipogenic and osteogenic potential differentiation as described below.

#### 2.4.1. Adipogenic Differentiation

PA20 cell line and PA20-h5 finite clonal line were cultured with a specific adipogenic medium (AM): in Ham's F12 Coon's modification medium supplemented with 10% (FBS), 100 IU/mL penicillin, 100 *μ*g/mL streptomycin and 1 *μ*M dexamethasone, 1 *μ*M bovine insulin, and 0.5 mM isobutylmethylxanthine (IBMX). The medium was refreshed twice a week. The expression of the adipogenic phenotype was evaluated on cells cultured in AM or GM for 35 days by Oil Red O staining.

#### 2.4.2. Osteogenic Differentiation

PA20 cell line and PA20-h5 finite clonal line were plated on tissue culture dishes at a cell density of 1 × 10^4^ cells/cm^2^ in GM and grown to 70–80% confluence. Afterwards, the medium was switched to osteogenic medium (OM): Ham's F12 Coon's modification medium supplemented with 10% FBS, 100 IU/mL penicillin, 100 *μ*g/mL streptomycin, 10 nM dexamethasone, 0.2 mM sodium L-ascorbyl-2-phosphate, and 10 mM *β*-glycerol phosphate. The medium was refreshed twice a week. The expression of the osteoblastic phenotype was evaluated at 15 and 30 days from induction by contemporary monitoring ALP activity and mineralization by cytochemical staining. For ALP staining, the cells were washed with DPBS (two times), stained with a specific dye mixture (5 mg naphthol-AS-MX phosphate sodium salt dissolved in 1 mL dimethyl sulfoxide), 40 mg Fast Red Violet LB dissolved in 49 mL Tris-HCl Buffer 280 mM pH 9.0 for 30 min at 37°C. Then, the cells were washed with DPBS (two times), fixed in 4% paraformaldehyde (PFA)/DPBS for 15 min, and washed with ultrapure water (three times). ALP+ cells were stained in red and nuclei were counterstained in blue with Mayer's acid hemalum. For mineralization staining, the cells were washed with DPBS (two times), fixed in 4% PFA/DPBS for 15 min, and washed with ultrapure water (three times). Calcium mineral deposits were stained for 2 min with 2% Alizarin Red S, pH 6.0, rinsed with water calcium mineralized deposits were stained in red-orange.

### 2.5. Treatment with Pharmacological Agents

The effects of treatment with Sr^2+^ on cell growth and osteogenic differentiation of PA20-h5 cells were evaluated at different concentrations and times from differentiation, depending on the parameters tested. In our study, we have used SrCl_2_ such as source of Sr^2+^. For the effects on cell growth, a range of concentrations of Sr^2+^ from 5 *μ*M to 400 *μ*M were tested, including 120 *μ*M, the average concentration of Sr^2+^ detectable in the serum of patients receiving the standard dose of 2 g/day of SR. For the analysis of ALP activity and* in vitro* mineralization, the concentrations of Sr^2+^ used ranged from 2.5 *μ*M to 400 *μ*M.

### 2.6. Analysis of Cell Proliferation in the Presence of Sr^2+^

PA20-h5 cells were seeded in 100 mm diameter dishes at a concentration of 20,000 cells/dish. After 24 hours, GM was replaced with Coon's medium added with 1.5% FCS and maintained in culture for 3 days. At the end, Coon's medium was replaced with Coon's medium with 1.5% FCS, without osteogenic induction factors, containing several different concentrations of Sr^2+^: 5, 50, 100, 200, and 400 *μ*M. The number of cells was evaluated at 0, 3, 6, 10, 13, and 16 days, the growth curves were plotted, and the cell population doubling time was calculated. Each experimental point was performed in triplicate, and each experiment was repeated three times.

### 2.7. Analysis of ALP and Calcium Mineralized Deposits Activity in Presence of Sr^2+^

The PA20-h5 clonal line was seeded in 24 multiwell plates at a concentration of 20,000 cells/well. At confluence, the GM was replaced with OM containing the fluorophore calcein 1 *μ*g/mL and different concentrations of Sr^2+^ from 2.5 *μ*M to 400 *μ*M and incubated from 7 to 35 days. At the end of the incubation, the cells were washed with DPBS (two times), fixed in 4% PFA/DPBS for 15 min, washed with ultrapure water (three times), dried, and preserved at 4°C until the assay. Each experimental point was performed in quadruplicate and each experiment was repeated three times.

#### 2.7.1. ALP Assay

Each well was incubated with 500 *μ*L of 4-methylumbelliferyl phosphate in 280 mM Tris-HCl Buffer pH 9.0 for 15 min at 37°C. The reaction was stopped by the addition of 2 mL 0.1 M NaOH. ALP activity was measured with a spectrofluorometer LS55 (PerkinElmer) at 365 nm *λ* excitation and 445 nm *λ* emission and expressed in *μ*U/cm^2^ using a standard curve of 4-methylumbelliferone 50 nM–10 *μ*M in 280 mM Tris-HCl Buffer pH 9.0.

#### 2.7.2. Calcium Mineralized Deposits Assay

Each well was incubated with 2 mL of 50 mM NaEDTA for 30 min at 37°C. The solution was then transferred into the cuvette and the fluorescence measured with a spectrofluorometer LS55 (PerkinElmer) at 494 nm *λ* excitation and 517 nm *λ* emission and expressed in *μ*g/cm^2^ using a standard curve of calcium mineralized deposits 25 ng/mL–500 *μ*g/mL solubilized in 50 mM NaEDTA.

### 2.8. Gene Expression Analysis

#### 2.8.1. Adipogenic Differentiation

The expression of the adipogenic phenotype in the PA20-h5 cell line was evaluated on cells cultured on PS in AM or GM for 21 days by RT-PCR analysis of the marker genes peroxisome proliferator-activated receptor 2 (*PPARγ2*) and lipoprotein lipase (*LPL*). At 21 days from induction cells were detached and sedimented for RNA extraction. Total RNA was extracted using RNAwiz RNA Isolation Reagent (Ambion, Inc., Austin, TX, USA), according to the manufacturer's protocol. The RNA was treated with DNA-free Kit (Ambion). Complementary first strand DNA (cDNA) was synthesized from 1 *μ*g of total RNA using ImProm-II Reverse Transcription System (Promega, Madison, WI, USA) and Oligo dT according to the manufacturer's protocol. The RT-PCR reactions of adipocytic* PPARγ2* and* LPL* genes were performed in triplicate, using *β-actin* as control. Reverse transcription products (2–5 *μ*L) were amplified in Bio-Rad iCycler system thermocycler (Bio-Rad Laboratories S.r.l., Segrate, Milan, Italy) using a 25 *μ*L reaction mixture containing 1 *μ*m of each primer and puRe Taq Ready-To-Go PCR Beads (Amersham Biosciences Corp., Piscataway, NJ, USA) with a standard thermal profile. Sequence, *T*_*m*_, and expected fragment size for each pair of primers are shown in Tables 1 and 2. The identity of each PCR product was confirmed by agarose gel electrophoresis and direct DNA sequencing using an ABI-Prism 3100 Genetic Analyzer (Applied Biosystem, Foster City, CA, USA).

#### 2.8.2. Osteogenic Differentiation

Gene expression analysis in the PA20-h5 cell line before and after 21 days in GM or OM was performed in the presence of 100 *μ*M Sr^2+^, a concentration known to be active on both cell proliferation and ALP enzymatic activity. The genes included in the analysis were* ALP* (known to be involved in the initial phases of the osteogenic differentiation),* RUNX2* (known as a precocious transcriptional factor during the osteoblastic differentiation), and* DKK1* (known to be an antagonist of osteoblastic differentiation).

Total RNA was extracted from frozen PA20-h5 cell pellets with Qiazol reagent (Qiagen) according to the manufacturer's instructions. Concentration, purity, and integrity of the total RNA were checked with an ND-1000 Spectrophotometer (NanoDrop Technologies, Wilmington, DE, USA) and also with an electrophoresis run on a 0.8% agarose gel. One microgram of total RNA was reverse-transcribed using QuantiTect Reverse Transcription Kit (Qiagen) according to manual instructions. To verify the successful reverse transcription, qualitative PCR was performed using 1 *μ*L cDNA as template and 10 *μ*M of each primer (forward and reverse) (Table 1) of the gene housekeeping *β*-actin. The genetic expression analysis by quantitative real time PCR (qRT-PCR) for* ALP*, runt-related transcription factor-2 (*RUNX2*),* DKK1*, and 40S ribosomal protein S18 (*RPS18*) was performed using Stratagene Mx3000-P Detection System (Stratagene, La Jolla, CA, USA). Reactions were carried out using a TaqMan 5′-exonuclease assay, following the thermic profile according to manual instructions (Kapa probe fast qPCR Kit, Kapa Biosystems). The primers and internal labelled oligonucleotides TaqMan probes for each cDNA, described in Table 2, were designed by IDT integrated DNA technologies. The cDNA samples used for the construction of standard curves for quantitative analysis were subjected to PCR amplification for each gene (primer sequences are indicated in Table 3), and PCR products were analyzed by 0.8% agarose gel electrophoresis visualized by ethidium-bromide staining and in the presence of marker VIII (Roche) performed by agarose gel elution band with Kit Millipore. The standard curves were generated by assessing serial cDNA dilutions (10-fold dilution for 8 logarithms) and plotting fluorescence versus the Ct (threshold cycle) based on dRn (baseline corrected, reference dye-normalized fluorescence). All points for standard curves and unknown samples were performed in triplicate. Negative control tubes with water were included in each real-time PCR run to detect any carry-over contamination. Target gene expression was normalized to* RPS18*.

### 2.9. Statistical Analysis

For proliferation analysis, statistical processing was performed during the “log phase” of the growth curves using (a) the linearity test by Student's *t*-test and the *R* ^2^ coefficient of determination for each regression and (b) the parallelism test by Student's *t*-test to compare growth curves in the presence of the different Sr^2+^ concentrations with the growth curve of the control. For ALP and calcium mineralized deposits assays the experiments were carried out in quadruplicate and each experiment was repeated three times. Gene expression analysis was performed in triplicate. All data were expressed as means ± S.D. Statistical differences among mean values were analyzed using Student's *t*-test.

## 3. Results

### 3.1. Cell Line Characterization

The PA20 cell line showed a doubling time of 67 days, while the PA20-h5 finite clonal cell line showed a doubling time of 56 days. The PA20-h5 line did not show growth in soft agar after 4 weeks in culture.

### 3.2. Adipogenic Differentiation

**Figure 1:**
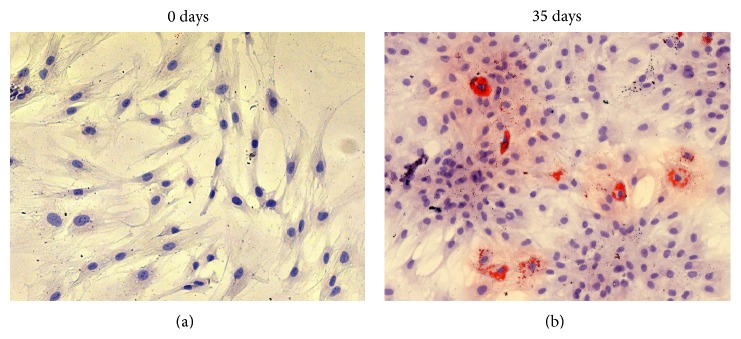
Observation in brightfield microscopy of PA20-h5 line after 0 days (a) and 35 (b) days of adipogenic induction. Cytochemical staining with Oil Red O; intracellular deposits of lipids stained in red; nuclei counterstained in blue-violet with hematoxylin (20x objective).

Adipogenic differentiation was not observed in the PA20-h5 line at time 0 (days), while after 35 days of adipogenic induction some cells showed intracellular vacuoles containing drops of lipids of variable shape and size (Figure 1). Similar results were observed in the primary PA20 cell line (data not shown).

Adipogenic differentiation was confirmed by RT-PCR of adipocyte-specific* LPL* and* PPARγ2* genes. In the absence of adipogenic induction, qualitative RT-PCR showed lack of expression for* PPARγ2* gene and a minuscule expression for* LPL* gene in the PA20-h5 line, while after 21 days from adipogenic induction, qualitative RT-PCR revealed a bright band on agarose gel for both genes (data not shown).

Adipogenic differentiation was not effected on SaOS-2 cell line, insofar as this line is already irrevocably directed in osteogenic sense.

### 3.3. Osteogenic Differentiation

#### 3.3.1. ALP Activity

**Figure 2:**
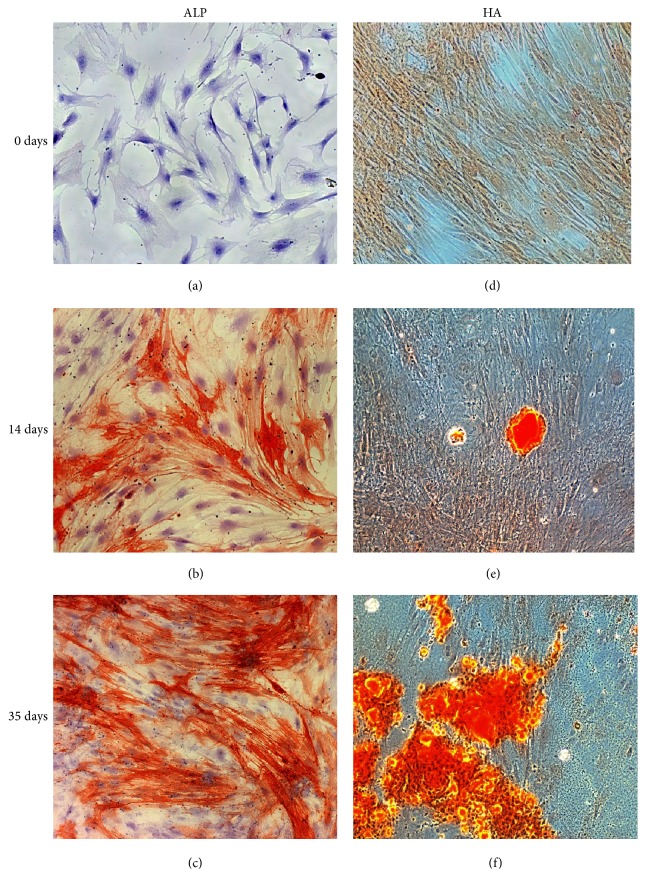
Observation in light microscopy of osteogenic differentiation of the PA20-h5 line at 0 days ((a), (d)), 14 days ((b), (e)), and 35 days of induction ((c), (f)). Cytochemical staining for ALP ((a), (b), and (c)): positive cells stained in red and nuclei counterstained in blue (observation in brightfield microscopy, 20x objective). Cytochemical staining for calcium mineralized deposits ((d), (e), and (f)): deposits stained in red-orange (observation in phase contrast microscopy, 20x objective).

PA20-h5 line did not show ALP activity at time 0 (days), while culture in the OM up to 35 days induced an increase in the number of cells positive to ALP that was time-dependent up to a maximum of approximately 40% of the cell population at 35 days (Figures 2(a), 2(b), and 2(c)). SaOS-2 cell line at only time 0 presents already 100% positivity, as expected for an osteoblast-like cell line, where all cells are already directed in osteogenic sense (data not shown).

#### 3.3.2. Calcium Mineralized Deposits

Results obtained showed a production of calcium mineralized deposits in the PA20-h5 line cultured with OM for 35 days. The number and size of the mineralized nodules were time-dependent. After 35 days, cell death and degeneration were observed near large mineralized deposits (Figures 2(d), 2(e), and 2(f)).

### 3.4. Analysis of Cell Proliferation in the Presence of Sr^2+^

**Figure 3:**
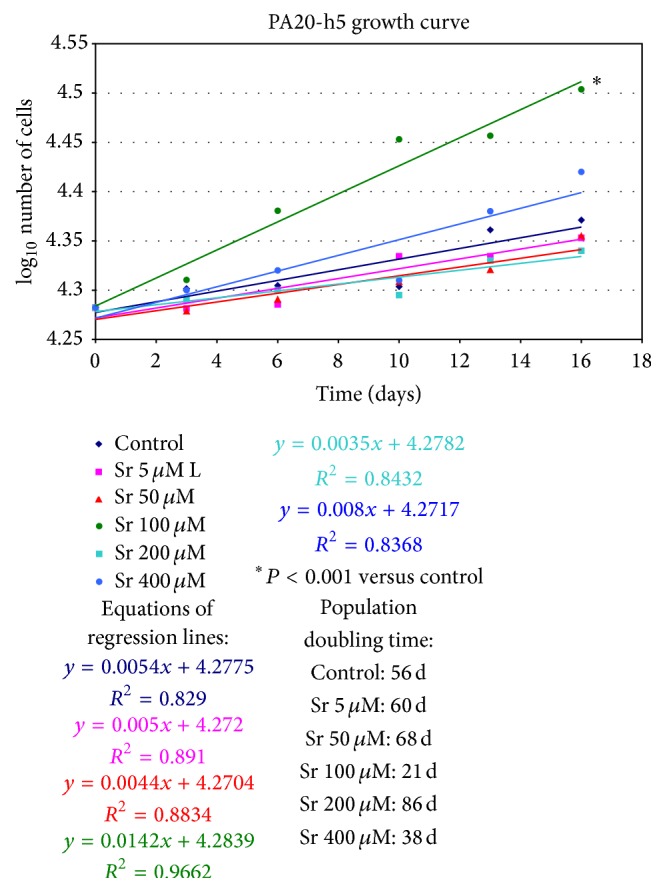
Graphical representation in linear regression of the kinetics of growth for PA20-h5 line cultured in Coon's medium + FCS 1.5% in the presence of Sr^2+^ concentrations from 0 to 400 *μ*M. The equation and the *R* ^2^ value are reported for each straight line. Experiments are carried out in triplicate and are representative of three different experiments.

Statistical processing performed during the “log phase” of the growth curves has shown the goodness of the linearity of the individual regressions (*P* < 0.001), with *R* ^2^ coefficient of determination, which always resulted greater than 0.80. The comparison between the linear regressions of the growth curves in presence of different Sr^2+^ concentrations and the control have shown significant differences (*P* < 0.001) for 100 *μ*M Sr^2+^ versus control with a respective doubling time of 21 days and 56 days. At lower and higher concentrations Sr^2+^ had no significative effects (Figure 3).

On SaOS-2 cell line was observed an analogous response with a significant increase versus control of the proliferative activity in the presence of 100 *μ*M Sr^2+^ (data not shown).

### 3.5. Quantitative Analysis of ALP Enzymatic Activity

**Figure 4:**
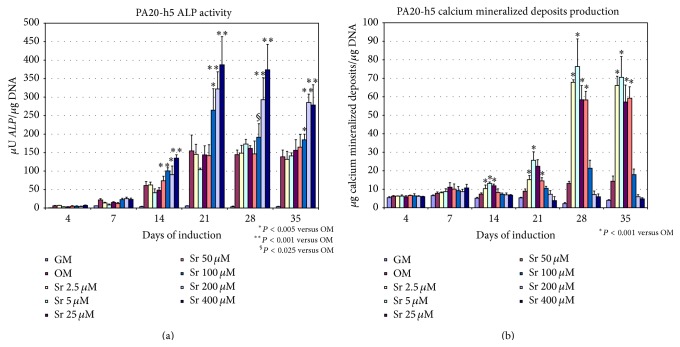
Quantitative analyses of ALP enzymatic activity (a) and calcium mineralized deposits (b) in PA20-h5 cultured in OM in the presence of scalar concentrations of strontium from 4 to 35 days. Experiments are carried out in triplicate and are representative of three different experiments.

At 100 *μ*M–400 *μ*M concentrations, Sr^2+^ significantly stimulated ALP production in the PA20-h5 cells, from 14 to 35 days, with maximal response being observed at 21 days with 400 *μ*M Sr^2+^ (150% versus control) and a decrease at longer times of observation (Figure 4(a)). Lower Sr^2+^ concentrations were inactive.

### 3.6. Quantitative Analysis of the Formation of Calcium Mineralized Deposits

A significant increase of HA production was observed compared to control from 14 to 35 days only at low Sr^2+^ doses (2.5 *μ*M–50 *μ*M). The maximal response was observed at 28 days for 5 *μ*M Sr^2+^ with an increase of 480% versus control. At higher concentrations, Sr^2+^ did not significantly affect HA production (Figure 4(b)).

Similarly, on SaOS-2 cell line the 5 *μ*M Sr^2+^ concentration seems to be the most effective between concentrations tested at 28 days with an increase from 14 to 35 days compared to control (data not shown).

### 3.7. Gene Expression Analysis of* ALP*,* RUNX2*, and* DKK1*

**Figure 5:**
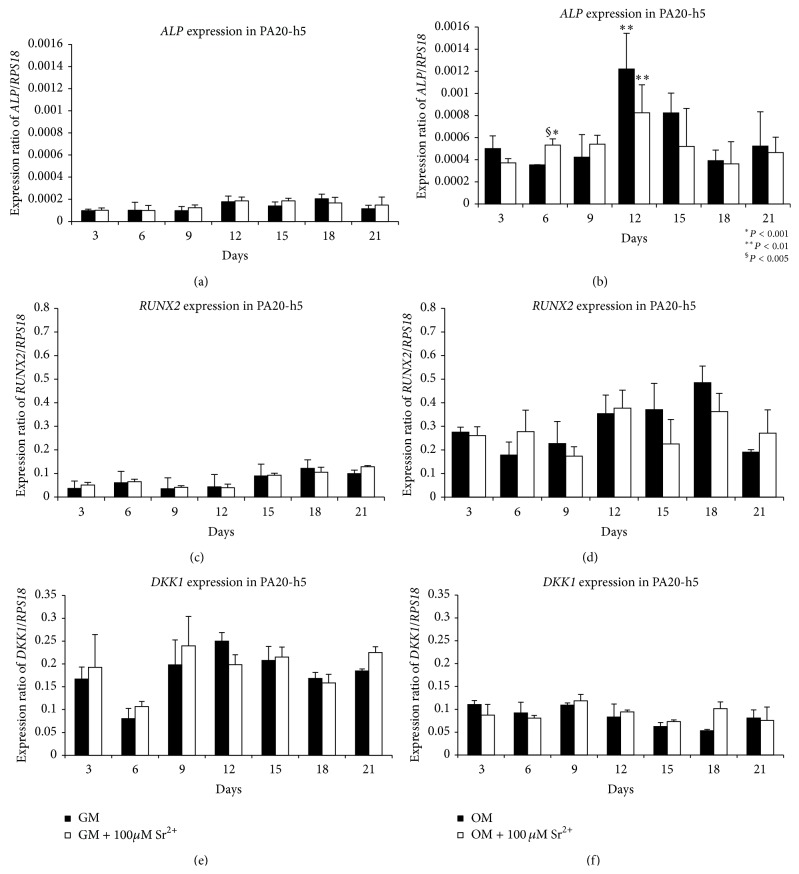
Analysis of gene expression of* ALP* ((a), (b)),* RUNX* ((c), (d)), and* DKK1* ((e), (f)) in PA20-h5 from 3 to 21 days. Culture in medium GM and GM containing 100 *μ*M Sr^2+^ ((a), (c) and (e)). Culture in medium OM and OM containing 100 *μ*M Sr^2+^ ((b), (d), and (f)). Experiments are carried out in triplicate and are representative of three different experiments.

Analysis of data showed the absence of significant variations of* ALP*,* RUNX2*, and* DKK1* gene expression in cells cultured in GM containing 100 *μ*M Sr^2+^ compared to those cultivated only in GM (Figures 5(a), 5(c), and 5(e)). However,* ALP* and* RUNX2* expression significantly increased in a time-dependent manner for both cells cultivated in OM and those cultivated in OM containing 100 *μ*M Sr^2+^, reaching a maximum for* ALP* at 12 days and for* RUNX2* at 18 days and both decreasing with time (Figures 5(b) and 5(d)). Significant increases of ALP expression were observed in cells cultured in OM containing 100 *μ*M Sr^2+^ at 6 days, both versus OM at 6 days (*P* < 0.005) and versus OM containing 100 *μ*M Sr^2+^ at 3 days (*P* < 0.001). Furthermore, significant increases of ALP expression were also observed in cells cultured in OM and in OM containing 100 *μ*M Sr^2+^ at 12 days, respectively, versus OM and OM containing 100 *μ*M Sr^2+^ at 3 days (*P* < 0.01) (Figure 5(b)). No variation of* DKK1* expression has been observed for cells cultivated in OM containing 100 *μ*M Sr^2+^ compared to those cultivated in OM (Figure 5(f)).

## 4. Discussion

Strontium ranelate has been shown in clinical trials to protect against bone loss and to reduce the risk of vertebral and hip fractures in postmenopausal women with osteoporosis [53, 54].

SR has a dual mode of action that differentiates it from other available treatments for osteoporosis. Indeed, it induces opposite effects on osteoclast and osteoblast* in vitro* cultures (i.e., enhances preosteoblast replication and collagen synthesis and inhibits osteoclast differentiation and resorbing activity by stimulating osteoclast apoptosis) [56]. These* in vitro* effects result* in vivo* in increased bone architecture and bone strength in studies on animal models [60–62, 67, 68]. In particular, in* in vivo* studies, on rat model treated with biomaterial Sr-enriched, the F18-fluoride PET analysis showed that the Sr released by biomaterial is incorporated in the mineralized matrix, promoting the bone healing [79].

In this paper, we describe the effect of Sr^2+^ on a novel adipose tissue-derived clonal human cell line, named PA20-h5. The PA20-h5 cell line shows a staminality feature, as confirmed by its differentiative capacity in both osteogenic and adipogenic lineages. This finding has important implications, as a human clonal cell line capable of expressing this phenotype has not been described before.

A wide range of Sr^2+^ concentrations was tested in PA20-h5 cell proliferation, ALP activity, and* in vitro* mineralization, showing the Sr^2+^ capability to promote both proliferation and osteoblastic differentiation.

The proliferative effects were seen at 100 *μ*M Sr^2+^ concentration, very close to the circulating doses of Sr^2+^ in patients treated with the standard dose 2 g/day of SR [53, 56].* In vitro* studies on rat calvarial cultures showed that SR at concentrations 0.01–1 mM and 5–10 mM enhanced the cell replication [62, 65].

*In vitro* osteodifferentiation of PA20-h5 cells monitored by ALP production was observed at 400 *μ*M Sr^2+^ concentration, supporting a role for Sr^2+^ in the early induction of bone differentiation. Indeed, a study on the mouse osteoblastic MC3T3-E1 cell line treated with 1 mM Sr^2+^ showed a significant increase of ALP activity at 4 and 14 days of culture compared with control [61].

At lower concentrations (2.5 to 50 *μ*M), Sr^2+^ stimulated HA production in the PA20-h5 cell line, with an opposite effect at higher concentrations (200 *μ*M and 400 *μ*M). This biphasic action indicates that low Sr^2+^ concentrations are capable of influencing the* in vitro* mineralization process. Altogether, the differential dose-dependent effects of Sr^2+^ on various steps of PA20-h5 osteoblastogenesis evidenced the complexity of the interaction of the cation bioeffects on the bone regeneration process. In fact, the higher doses (200 *μ*M and 400 *μ*M) of Sr^2+^ in the PA20-h5 seem to inhibit the formation of HA deposits presupposing alterations of the physicochemical properties in the structure of hydroxyapatite crystal up to impede its formation [80, 81].

The increase in ALP production was confirmed by gene expression results in which 100 *μ*M Sr^2+^ induced an increase in* ALP* gene expression in PA20-h5 osteoinduced cells, with no effect on* RUNX2* and* DKK1* gene expression. Based on these results, Sr^2+^ seems to act early on the* in vitro* osteogenic induction of the PA20-h5 cell line. These observations do not seem to confirm previous findings in other cellular models. Indeed, an* in vitro* study on rat BMMSCs has shown that SR at concentrations of 0,1, and 1 mM Sr^2+^ promotes the osteoblastic differentiation both by increase of ALP expression and by mRNA levels of RUNX2, bone sialoprotein, and osteocalcin, while it significantly inhibits proliferation [75]. Another* in vitro* study showed induction of early expression of* RUNX2* at day 4 by Sr^2+^ in hMSCs [78]. In addition, it was demonstrated that 4 mM Sr^2+^ increased mRNA expression of* RUNX2* and osteocalcin (*OCN*) in hMSCs* in vitro* [82].

Finally, in primary osteoblasts derived from mouse calvaria, 0.1–1 mM SR concentrations promoted bone nodule formation, increasing the differentiation from early progenitor cells to mature osteoblasts, as reflected also by the increase of the expression of osteoblastic markers such as ALP, bone sialoprotein (*BSP*), and* OCN* [66]. In another* in vitro* study, it was instead seen that, in U-33 preosteoblastic cells, 0.1–1 mM Sr^2+^ concentrations significantly enhanced the expression of* RUNX2* and* OCN* genes, but not* BSP*, while in more mature osteoblastic OB-6 cells Sr^2+^ induced only minimal effects on* RUNX2* expression, but presented a positive effect on* BPS* and* OCN* expression [66].

The differences in the results obtained encompass both sensitivity to Sr^2+^ challenge and qualitative responses. The reason for such variability could be explained by the diverse cellular models used up to now, which include rodent versus human cell cultures and clonal versus mixed cellular models. It seems, therefore, reasonable to address future research in this area using human models and continuous cell lines. This will make it possible to avoid heterogeneity in the response to Sr^2+^.

## 5. Conclusions

Multiple molecular and biological mechanisms are involved in cell proliferation and osteogenic differentiation* in vitro* and* in vivo*. The present study showed how different concentrations of Sr^2+^ act on hADSCs depending on the biological phenomenon analyzed. On the basis of our results,* in vitro* Sr^2+^ ion treatment of hADSCs enhances cell proliferation and osteogenic differentiation through expression of early and late osteoblastic biomarkers such as ALP and HA, respectively. This effect is dose-dependent, with a positive effect at circulating pharmacological Sr^2+^ doses. These findings clearly support the use of SR in* in vitro* induction of bone regeneration. Future studies will try to answer fundamental questions regarding the use of SR treatment in patients undergoing cell therapy and administration.

## Figures and Tables

**Table 1:** Primers used for the *β*-actin gene.

Gene	Primer sequence (5′-3′)	Amplicon size (bp)	*T*_*m*_ (°C)
*β*-actin FOR *β*-actin REV	GACCTGACTGACTACCTCATGAA CTTCATGATGGAGTTGAAGGTA	303	60

**Table 2:** Primers used for RT-PCR.

Gene	Primer sequences (5′-3′)	Amplicon size (bp)	*T*_*m*_ (°C)
*PPAR-γ2* FOR *PPAR-γ2* REV	AGGAGCAGAGCAAAGAGG CCTCGGATATGAGAACCC	219	58

*LPL* FOR *LPL* REV	AGTTGTACTTCCAGTGCGTCTC TACTTTCACAGTCGGGTT	366	56

bp, base pairs of amplicon size; *T*_*m*_ (°C), melting temperature (°C).

**Table 3:** Primers and TaqMan probes used for qRT-PCR.

Gene	Primer sequences (5′-3′) and TaqMan probes	Amplicon size (bp)	*T*_*m*_ (°C)
***RPS18*** FOR ***RPS18*** REV **probe**	TCTTCCACAGGAGGCCTAC GATGGCAAAGGCTATTTTCCG **F/**TTCAGGGAT/**ZEN**/CACTAGAGACATGGCTGC/**Q**	132	60

***ALP195*** FOR ***ALP195*** REV **probe**	CCCGTGGCAACTCTATCTTTG CATACAGGATGGCAGTGAAGG **F/**TTCTTGTCT/**ZEN**/GTGTCACTCAGCATGGG/**Q**	78	60

***DKK1*** FOR ***DKK1*** REV **probe**	TGATCATAGCACCTTGGATGG ACACAATCCTGAGGCACAG **F**/CTGATGACC/**ZEN**/GGAGACAAACAGAACCTT/**Q**	121	60

***RUNX2*** FOR ***RUNX2*** REV **probe**	AGGGACTATGGCATCAAACAG CTTCACGTCGCTCATTTTGC **F**/TCTTTTGGA/**ZEN**/TCCGAGCACCAGCC/**Q**	135	60

TaqMan probes with F as reporter fluorochrome (6-carboxyfluorescein [6-FAM]) and Q as quencher.

Fluorochrome (Iowa Black FQ); bp, base paris of amplicon size; *T*_*m*_, melting temperature (°C).

## References

[1] Burdick J. A., Mason M. N., Hinman A. D., Thorne K., Anseth K. S. (2002). Delivery of osteoinductive growth factors from degradable PEG hydrogels influences osteoblast differentiation and mineralization. *Journal of Controlled Release*.

[2] Garg N. K., Gaur S., Sharma S. (1993). Percutaneous autogenous bone marrow grafting in 20 cases of ununited fracture. *Acta Orthopaedica Scandinavica*.

[3] Garg N. K., Gaur S. (1995). Percutaneous autogenous bone-marrow grafting in congenital tibial pseudarthrosis. *The Journal of Bone & Joint Surgery—British Volume*.

[4] Healey J. H., Zimmerman P. A., McDonnell J. M., Lane J. M. (1990). Percutaneous bone marrow grafting of delayed union and nonunion in cancer patients. *Clinical Orthopaedics and Related Research*.

[5] Kadiyala S., Young R. G., Thiede M. A., Bruder S. P. (1997). Culture expanded canine mesenchymal stem cells possess osteochondrogenic potential *in vivo* and *in vitro*. *Cell Transplantation*.

[6] Ringe J., Kaps C., Burmester G.-R., Sittinger M. (2002). Stem cells for regenerative medicine: advances in the engineering of tissues and organs. *Naturwissenschaften*.

[7] Kwan M. D., Slater B. J., Wan D. C., Longaker M. T. (2008). Cell-based therapies for skeletal regenerative medicine. *Human Molecular Genetics*.

[8] Jorgensen C., Gordeladze J., Noel D. (2004). Tissue engineering through autologous mesenchymal stem cells. *Current Opinion in Biotechnology*.

[9] Tognarini I., Sorace S., Zonefrati R. (2008). *In vitro* differentiation of human mesenchymal stem cells on Ti6Al4V surfaces. *Biomaterials*.

[10] Bauer T. W., Muschler G. F. (2000). Bone graft materials: an overview of the basic science. *Clinical Orthopaedics and Related Research*.

[11] Fleming J. E., Cornell C. N., Muschler G. F. (2000). Bone cells and matrices in orthopedic tissue engineering. *Orthopedic Clinics of North America*.

[12] Hicok K. C., Du Laney T. V., Zhou Y. S. (2004). Human adipose-derived adult stem cells produce osteoid *in vivo*. *Tissue Engineering*.

[13] Bianco P., Riminucci M., Gronthos S., Robey P. G. (2001). Bone marrow stromal stem cells: nature, biology, and potential applications. *Stem Cells*.

[14] Zuk P. A., Zhu M., Mizuno H. (2001). Multilineage cells from human adipose tissue: implications for cell-based therapies. *Tissue Engineering*.

[15] Dawn B., Bolli R. (2005). Adult bone marrow-derived cells: regenerative potential, plasticity, and tissue commitment. *Basic Research in Cardiology*.

[16] Gimble J. M., Guilak F. (2003). Adipose-derived adult stem cells: isolation, characterization, and differentiation potential. *Cytotherapy*.

[17] Zuk P. A., Zhu M., Ashjian P. (2002). Human adipose tissue is a source of multipotent stem cells. *Molecular Biology of the Cell*.

[18] Gimble J. M., Katz A. J., Bunnell B. A. (2007). Adipose-derived stem cells for regenerative medicine. *Circulation Research*.

[19] Planat-Benard V., Silvestre J.-S., Cousin B. (2004). Plasticity of human adipose lineage cells toward endothelial cells: physiological and therapeutic perspectives. *Circulation*.

[20] Safford K. M., Hicok K. C., Safford S. D. (2002). Neurogenic differentiation of murine and human adipose-derived stromal cells. *Biochemical and Biophysical Research Communications*.

[21] Seo M. J., Suh S. Y., Bae Y. C., Jung J. S. (2005). Differentiation of human adipose stromal cells into hepatic lineage *in vitro* and *in vivo*. *Biochemical and Biophysical Research Communications*.

[22] Huang J. I., Zuk P. A., Jones N. F. (2004). Chondrogenic potential of multipotential cells from human adipose tissue. *Plastic and Reconstructive Surgery*.

[23] Zhao Y., Lin H., Zhang J. (2009). Crosslinked three-dimensional demineralized bone matrix for the adipose-derived stromal cell proliferation and differentiation. *Tissue Engineering Part A*.

[24] Lee J. H., Rhie J. W., Oh D. Y., Ahn S. T. (2008). Osteogenic differentiation of human adipose tissue-derived stromal cells (hASCs) in a porous three-dimensional scaffold. *Biochemical and Biophysical Research Communications*.

[25] Gonzalez-Rey E., Gonzalez M. A., Varela N. (2010). Human adipose-derived mesenchymal stem cells reduce inflammatory and T cell responses and induce regulatory T cells in vitro in rheumatoid arthritis. *Annals of the Rheumatic Diseases*.

[26] Gonzalez-Rey E., Anderson P., González M. A., Rico L., Büscher D., Delgado M. (2009). Human adult stem cells derived from adipose tissue protect against experimental colitis and sepsis. *Gut*.

[27] Meza-Zepeda L. A., Noer A., Dahl J. A., Micci F., Myklebost O., Collas P. (2008). High-resolution analysis of genetic stability of human adipose tissue stem cells cultured to senescence: stem cells. *Journal of Cellular and Molecular Medicine*.

[28] Dahl J. A., Duggal S., Coulston N. (2008). Genetic and epigenetic instability of human bone marrow mesenchymal stem cells expanded in autologous seum or fatal bovine serum. *International Journal of Developmental Biology*.

[29] Gronthos S., Franklin D. M., Leddy H. A., Robey P. G., Storms R. W., Gimble J. M. (2001). Surface protein characterization of human adipose tissue-derived stromal cells. *Journal of Cellular Physiology*.

[30] Witkowska-Zimny M., Walenko K. (2011). Stem cells from adipose tissue. *Cellular and Molecular Biology Letters*.

[31] Zhu Y., Liu T., Song K., Fan X., Ma X., Cui Z. (2008). Adipose-derived stem cell: a better stem cell than BMSC. *Cell Biochemistry and Function*.

[32] Tremolada C., Palmieri G., Ricordi C. (2010). Adipocyte transplantation and stem cells: plastic surgery meets regenerative medicine. *Cell Transplantation*.

[33] Kilroy G. E., Foster S. J., Wu X. (2007). Cytokine profile of human adipose-derived stem cells: expression of angiogenic, hematopoietic, and pro-inflammatory factors. *Journal of Cellular Physiology*.

[34] Lindroos B., Suuronen R., Miettinen S. (2011). The potential of adipose stem cells in regenerative medicine. *Stem Cell Reviews and Reports*.

[35] Fang B., Song Y., Liao L., Zhang Y., Zhao R. C. (2007). Favorable response to human adipose tissue-derived mesenchymal stem cells in steroid-refractory acute graft-versus-host disease. *Transplantation Proceedings*.

[36] Fang B., Song Y., Lin Q. (2007). Human adipose tissue-derived mesenchymal stromal cells as salvage therapy for treatment of severe refractory acute graft-vs.-host disease in two children. *Pediatric Transplantation*.

[37] Fang B., Song Y., Zhao R. C., Han Q., Lin Q. (2007). Using human adipose tissue-derived mesenchymal stem cells as salvage therapy for hepatic graft-versus-host disease resembling acute hepatitis. *Transplantation Proceedings*.

[38] Mesimäki K., Lindroos B., Törnwall J. (2009). Novel maxillary reconstruction with ectopic bone formation by GMP adipose stem cells. *International Journal of Oral and Maxillofacial Surgery*.

[39] Garcia-Olmo D., Garcia-Arranz M., Herreros D. (2008). Expanded adipose-derived stem cells for the treatment of complex perianal fistula including Crohn's disease. *Expert Opinion on Biological Therapy*.

[40] Riordan N. H., Ichim T. E., Min W.-P. (2009). Non-expanded adipose stromal vascular fraction cell therapy for multiple sclerosis. *Journal of Translational Medicine*.

[41] Yoshimura K., Sato K., Aoi N., Kurita M., Hirohi T., Harii K. (2008). Cell-assisted lipotransfer for cosmetic breast augmentation: supportive use of adipose-derived stem/stromal cells. *Aesthetic Plastic Surgery*.

[42] Yoshimura K., Sato K., Aoi N. (2008). Cell-assisted lipotransfer for facial lipoatrophy: efficacy of clinical use of adipose-derived stem cells. *Dermatologic Surgery*.

[43] Harris S. T., Watts N. B., Genant H. K. (1999). Effects of risedronate treatment on vertebral and nonvertebral fractures in women with postmenopausal osteoporosis: a randomized controlled trial. *The Journal of the American Medical Association*.

[44] Chesnut C. H., Skag A., Christiansen C. (2004). Effects of oral ibandronate administered daily or intermittently on fracture risk in postmenopausal osteoporosis. *Journal of Bone and Mineral Research*.

[45] Bilezikian J. P. (2009). Efficacy of bisphosphonates in reducing fracture risk in postmenopausal osteoporosis. *American Journal of Medicine*.

[46] Ettinger B., Black D. M., Mitlak B. H. (1999). Reduction of vertebral fracture risk in postmenopausal women with osteoporosis treated with raloxifene: results from a 3-year randomized clinical trial. *The Journal of the American Medical Association*.

[47] Ensrud K. E., Stock J. L., Barrett-Connor E. (2008). Effects of raloxifene on fracture risk in postmenopausal women: the raloxifene use for the heart trial. *Journal of Bone and Mineral Research*.

[48] Sugimoto T. (2011). Osteoporosis treatment by anti-RANKL antibody. *Clinical Calcium*.

[49] Adler R. A., Gill R. S. (2011). Clinical utility of denosumab for treatment of bone loss in men and women. *Clinical Interventions in Aging*.

[50] Reginster J. Y. (2011). Antifracture efficacy of currently available therapies for postmenopausal osteoporosis. *Drugs*.

[51] Neer R. M., Arnaud C. D., Zanchetta J. R. (2001). Effect of parathyroid hormone (1–34) on fractures and bone mineral density in postmenopausal women with osteoporosis. *The New England Journal of Medicine*.

[52] Roux C., Fechtenbaum J., Kolta S., Isaia G., Cannata Andia J. B., Devogelaer J.-P. (2008). Strontium ranelate reduces the risk of vertebral fracture in young postmenopausal women with severe osteoporosis. *Annals of the Rheumatic Diseases*.

[53] Meunier P. J., Roux C., Seeman E. (2004). The effects of strontium ranelate on the risk of vertebral fracture in women with postmenopausal osteoporosis. *The New England Journal of Medicine*.

[54] Reginster J. Y., Seeman E., de Vernejoul M. C. (2005). Strontium ranelate reduces the risk of nonvertebral fractures in postmenopausal women with osteoporosis: Treatment of Peripheral Osteoporosis (TROPOS) study. *The Journal of Clinical Endocrinology & Metabolism*.

[55] Marie P. J. (2006). Strontium ranelate: a dual mode of action rebalancing bone turnover in favour of bone formation. *Current Opinion in Rheumatology*.

[56] Marie P. J., Ammann P., Boivin G., Rey C. (2001). Mechanisms of action and therapeutic potential of strontium in bone. *Calcified Tissue International*.

[57] Marie P. J. (2005). Strontium ranelate: a novel mode of action optimizing bone formation and resorption. *Osteoporosis International*.

[58] Dahl S. G., Allain P., Marie P. J. (2001). Incorporation and distribution of strontium in bone. *Bone*.

[59] Bruyere O., Roux C., Detilleux J. (2007). Relationship between bone mineral density changes and fracture risk reduction in patients treated with strontium ranelate. *Journal of Clinical Endocrinology and Metabolism*.

[60] Marie P. J., Hott M., Modrowski D. (1993). An uncoupling agent containing strontium prevents bone loss by depressing bone resorption and maintaining bone formation in estrogen-deficient rats. *Journal of Bone and Mineral Research*.

[61] Barbara A., Delannoy P., Denis B. G., Marie P. J. (2004). Normal matrix mineralization induced by strontium ranelate in MC3T3-E1 osteogenic cells. *Metabolism*.

[62] Canalis E., Hott M., Deloffre P., Tsouderos Y., Marie P. J. (1996). The divalent strontium salt S12911 enhances bone cell replication and bone formation *in vitro*. *Bone*.

[63] Bonnelye E., Chabadel A., Saltel F., Jurdic P. (2008). Dual effect of strontium ranelate: stimulation of osteoblast differentiation and inhibition of osteoclast formation and resorption in vitro. *Bone*.

[64] Brennan T. C., Rybchyn M. S., Green W., Atwa S., Conigrave A. D., Mason R. S. (2009). Osteoblasts play key roles in the mechanisms of action of strontium ranelate. *British Journal of Pharmacology*.

[65] Chattopadhyay N., Quinn S. J., Kifor O., Ye C., Brown E. M. (2007). The calcium-sensing receptor (CaR) is involved in strontium ranelate-induced osteoblast proliferation. *Biochemical Pharmacology*.

[66] Zhu L.-L., Zaidi S., Peng Y. (2007). Induction of a program gene expression during osteoblast differentiation with strontium ranelate. *Biochemical and Biophysical Research Communications*.

[67] Baron R., Tsouderos Y. (2002). *In vitro* effects of S12911-2 on osteoclast function and bone marrow macrophage differentiation. *European Journal of Pharmacology*.

[68] Takahashi N., Sasaki T., Tsouderos Y., Suda T. (2003). S 12911-2 inhibits osteoclastic bone resorption *in vitro*. *Journal of Bone and Mineral Research*.

[69] Hurtel-Lemaire A. S., Mentaverri R., Caudrillier A. (2009). The calcium-sensing receptor is involved in strontium ranelate-induced osteoclast apoptosis. New insights into the associated signaling pathways. *The Journal of Biological Chemistry*.

[70] Marie P. J. (2007). Strontium ranelate: new insights into its dual mode of action. *Bone*.

[71] Fonseca J. E. (2008). Rebalancing bone turnover in favour of formation with strontium ranelate: implications for bone strength. *Rheumatology*.

[72] Atkins G. J., Welldon K. J., Halbout P., Findlay D. M. (2009). Strontium ranelate treatment of human primary osteoblasts promotes an osteocyte-like phenotype while eliciting an osteoprotegerin response. *Osteoporosis International*.

[73] Yang F., Yang D., Tu J., Zheng Q., Cai L., Wang L. (2011). Strontium enhances osteogenic differentiation of mesenchymal stem cells and *in vivo* bone formation by activating Wnt/catenin signaling. *Stem Cells*.

[74] Li Z., Wang Y., Wang X.-N., Lan A.-P., Wu W. (2011). Strontium ranelate promotes osteogenic differentiation of rat bone marrow mesenchymal stem cells by increasing bone morphogenetic protein-7 expression. *Nan fang Yi Ke Da Xue Xue Bao*.

[75] Li Y., Li J., Zhu S. (2012). Effects of strontium on proliferation and differentiation of rat bone marrow mesenchymal stem cells. *Biochemical and Biophysical Research Communications*.

[76] Choudhary S., Halbout P., Alander C., Raisz L., Pilbeam C. (2007). Strontium ranelate promotes osteoblastic differentiation and mineralization of murine bone marrow stromal cells: Involvement of prostaglandins. *Journal of Bone and Mineral Research*.

[77] Yang F., Tu J., Yang D., Li G., Cai L., Wang L. (2010). Osteogenic differentiation of mesenchymal stem cells could be enhanced by strontium. *Conference Proceedings: Annual International Conference of the IEEE Engineering in Medicine and Biology Society*.

[78] Sila-Asna M., Bunyaratvej A., Maeda S., Kitaguchi H., Bunyaratavej N. (2007). Osteoblast differentiation and bone formation gene expression in strontium-inducing bone marrow mesenchymal stem cell. *Kobe Journal of Medical Sciences*.

[79] Cheng C., Alt V., Pan L. (2014). Preliminary evaluation of different biomaterials for defect healing in an experimental osteoporotic rat model with dynamic PET-CT (dPET-CT) using F-18-Sodium Fluoride (NaF). *Injury*.

[80] Verberckmoes S. C., Behets G. J., Oste L. (2004). Effects of strontium on the physicochemical characteristics of hydroxyapatite. *Calcified Tissue International*.

[81] Verberckmoes S. C., de Broe M. E., D'Haese P. C. (2003). Dose-dependent effects of strontium on osteoblast function and mineralization. *Kidney International*.

[82] Peng S., Liu X. S., Wang T. (2010). *In vivo* anabolic effect of strontium on trabecular bone was associated with increased osteoblastogenesis of bone marrow stromal cells. *Journal of Orthopaedic Research*.

